# A205 IMPACT OF THE PANDEMIC ON BUDESONIDE MMX DISPENSING AND TREATMENT FAILURE IN ULCERATIVE COLITIS

**DOI:** 10.1093/jcag/gwae059.205

**Published:** 2025-02-10

**Authors:** S Coward, K Martins, S Klarenbach, K Kroeker, C Ma, R Panaccione, L Richer, C Seow, L Targownik, G G Kaplan

**Affiliations:** University of Calgary, Calgary, AB, Canada; University of Alberta, Edmonton, AB, Canada; University of Alberta, Edmonton, AB, Canada; University of Alberta, Edmonton, AB, Canada; University of Calgary, Calgary, AB, Canada; University of Calgary, Calgary, AB, Canada; University of Alberta, Edmonton, AB, Canada; University of Calgary, Calgary, AB, Canada; University of Toronto, Toronto, ON, Canada; University of Calgary, Calgary, AB, Canada

## Abstract

**Background:**

Budesonide MMX is a locally acting corticosteroid with reduced side effects compared to prednisone. During the pandemic, budesonide MMX may have been preferred due to its favourable side effect profile.

**Aims:**

To analyze rates of budesonide MMX dispensing and treatment failure before / during the pandemic.

**Methods:**

We analyzed population-based administrative healthcare data from Alberta to identify people dispensed budesonide MMX who met three criteria: 1. incident cases of ulcerative colitis (UC) after January 1, 2018; 2. ≥18 years old; 3. not concurrently on prednisone. We defined failure of budesonide MMX as subsequent prednisone dispensing within 30 days. We calculated rates of budesonide MMX dispensing per 1000 incident UC cases. We calculated average monthly percentage change (AMPC) in dispensing and failure rates with 95% confidence intervals (CI) by Poisson or negative binomial models. An interaction term tested differences between pre-pandemic rates (prior to April 2020) and pandemic rates (April 2020 to March 2023). We used Cox proportional hazard models to compare budesonide MMX failure before vs during the pandemic, with hazard ratios (HR) and 95%CIs—a sensitivity analysis was done to extend the follow-up time to 90 days.

**Results:**

Overall, 320 incident UC cases were dispensed budesonide MMX with 30-days follow-up. Of those, 14.06% (95%CI: 10.25, 17.87) received prednisone within 30 days. Mean time to failure was 17 days. There was a significant difference in the AMPC in dispensing rates was observed pre-pandemic vs during the pandemic (*p*=0.037) (Table 1). After the pandemic onset, budesonide MMX dispensing significantly decreased (AAPC: −2.43%; 95%CI: −3.77, −1.08). For the 30 days, the HR for budesonide MMX failure was 1.92 (95%CI: 0.95, 3.88). When the follow-up was extended to 90 days the HR was significant at 1.68 (95%CI: 1.05, 2.71), suggesting a significantly increased hazard of budesonide MMX failure during the pandemic compared to pre-pandemic.

**Conclusions:**

Budesonide MMX dispensing was stable pre-pandemic but significantly decreased during. The pandemic may have been associated with an increased failure rate of budesonide MMX—particularly within 90 days—as more patients required prednisone to manage their UC.

Analysis of Rates per 1000 Individuals with Incident UC

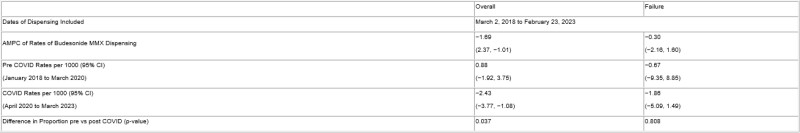

**Funding Agencies:**

Ferring Pharmaceuticals

